# Epithelial Downgrowth after Intraocular Surgery Treated with Intracameral 5-Fluorouracil

**DOI:** 10.1155/2015/325485

**Published:** 2015-05-28

**Authors:** Nina Ni, Marc A. Goldberg, Ralph C. Eagle, Christopher J. Rapuano, Julia A. Haller

**Affiliations:** ^1^Wills Eye Hospital, Thomas Jefferson University, Philadelphia, PA 19107, USA; ^2^The Eye Institute, Tulsa, OK, USA

## Abstract

*Purpose*. To present the clinical and histopathologic correlation of two cases of epithelial downgrowth (EDG) after prior intraocular surgery. *Methods*. Observational case reports. *Results*. We present two cases of EDG occurring after intraocular surgery. In both cases, after two anterior chamber injections of 5-fluorouracil (5FU), the area of EDG initially regressed. In Case 1, a limited area of EDG eventually recurred, and penetrating keratoplasty with cryotherapy was curative. In Case 2, subsequent corneal edema required Descemet-stripping automated endothelial keratoplasty, and the patient remained clinically free of EDG without further treatment. *Conclusion*. Intracameral 5FU may have a role in the treatment of EDG after intraocular surgery, though its precise utilization and impact remain to be defined.

## 1. Introduction

Epithelial downgrowth (EDG) is a serious complication of intraocular surgery and trauma [1–6]. Due to the high morbidity of traditional ablative surgical therapy [7–9], alternative treatment for anterior segment EDG has been investigated, including the chemotherapy agent 5-fluorouracil (5FU) [10–14]. We report two cases of intracameral 5FU-treated EDG with histopathologic correlation.

## 2. Case 1

**Figure 1:**
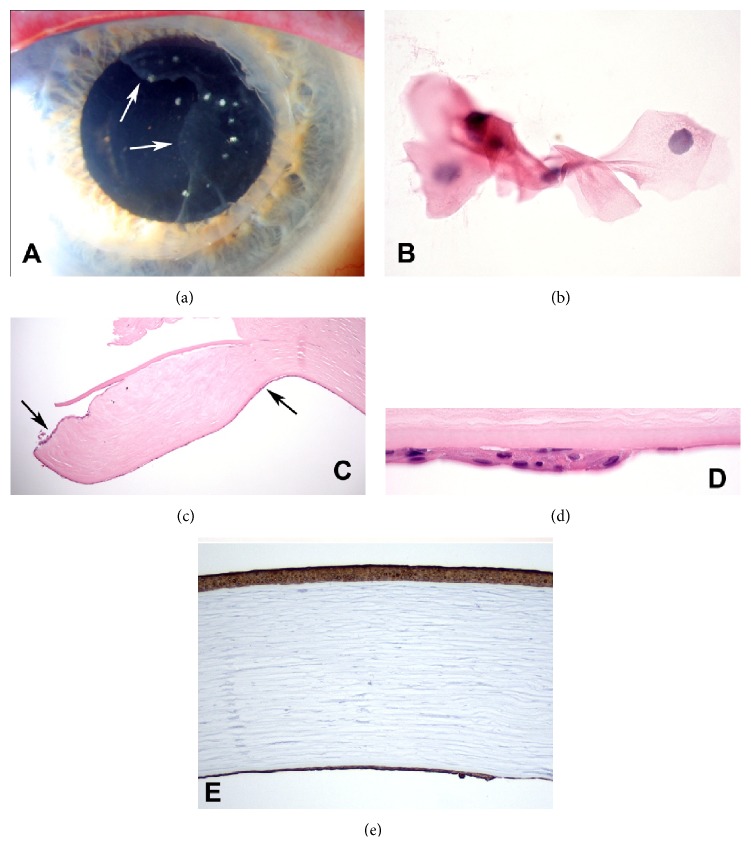
(a) Slit lamp photograph of the right eye showing epithelial downgrowth (EDG) involving the nasal aspect of the patient's graft (border indicated by arrows) and extending onto the superonasal native corneal endothelium. (b) Anterior chamber fluid aspirate with endothelial scraping shows the clump of squamous cells that made the diagnosis of EDG. (c) Periphery of penetrating keratoplasty specimen showing thin layer of surface epithelium (single arrow) on posterior surface of DSAEK graft. Epithelium has extended onto anterior surface of graft at left. (d) Margin of surface epithelial sheet on posterior surface of DSAEK graft. (e) Corneal epithelium and EDG on posterior cornea show similar intense immunoreactivity for cytokeratin marker AE1/AE3. (b) H&E ×400, (c) H&E ×25, (d) H&E ×250, and (e) IHC for AE1/AE3, ×50.

A 65-year-old man with Fuchs' corneal dystrophy underwent cataract surgery and subsequent uneventful Descemet-stripping automated endothelial keratoplasty (DSAEK) in the right eye (OD). After initially achieving 20/30 vision over postoperative months 2 through 9, his vision dropped to 20/60 at the 14-month visit. Slit lamp examination revealed a scalloped border of apparent EDG involving the nasal 20% of the graft, extending onto the superonasal native corneal endothelial surface (Figure 1). Diagnostic focal argon laser testing of the iris surface (250-mW, 100-*μ*m spot size, and 0.1-second duration) demonstrated no EDG involvement; if a membrane were present, the laser spots would lead to blanching. Cytology performed on an anterior chamber fluid aspirate with endothelial scrapings established the diagnosis of EDG (Figure 1).

After a discussion of treatment options, the patient decided to undergo anterior chamber 5FU injections in the operating suite. 5FU in a concentration of 1000 mcg/0.1 mL was mixed with 0.1 mL of viscoelastic (Viscoat, Alcon, Fort Worth, TX). After paracentesis with release of anterior chamber fluid and introduction of filtered air for visualization, the entire mixture was injected towards the area of EDG via 27-gauge needle. Balanced salt solution was then exchanged for the filtered air with care not to disrupt the 5FU-viscoelastic plug, and subconjunctival injections of ceftazidime and dexamethasone were delivered. An identical procedure was performed one week later. After resolution of initial corneal edema and an epithelial defect, the EDG resolved, and visual acuity was 20/60 at one month postoperatively. Over the subsequent 2 months, however, the patient developed progressive corneal edema, with vision declining to 20/200, and areas of corneal haze on slit lamp examination were suspicious for recurrent EDG. Penetrating keratoplasty (PKP) with cryotherapy to the area in the native cornea concerning for EDG was performed.

The PKP specimen and native corneal endothelial scrapings were sent for histopathologic analysis. On one side of the corneal specimen, the posterior surface of the endothelial graft was covered by a multilayered sheet of epithelial cells, consistent with EDG. Intense immunoreactivity for cytokeratin marker AE1/AE3 confirmed the diagnosis (Figure 1).

Postoperatively, the patient remained stable with visual acuity of 20/25 and a clear graft without recurrent EDG at 30 months.

## 3. Case 2

**Figure 2:**
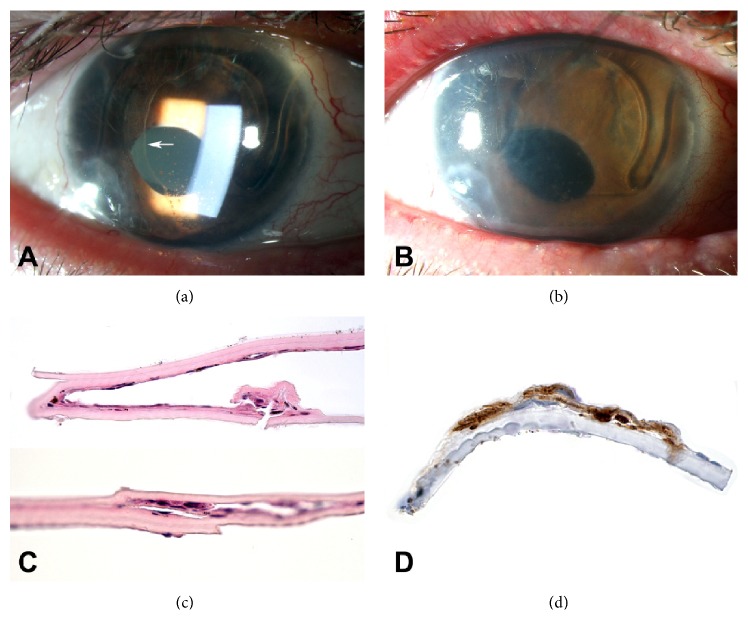
(a) Slit lamp photograph of the right eye showing epithelial downgrowth (EDG) involving the temporal aspect of the cornea (border indicated by arrow). (b) Four months after the 5FU injections, there was no clinical evidence of EDG, but there was significant corneal edema secondary to endothelial decompensation. (c) DSAEK specimen showing rare epithelial cells on the posterior surface of host cornea. (d) EDG on the posterior surface of a portion of the DSAEK graft shows immunoreactivity for cytokeratin marker AE1/AE3. (c) H&E ×50, (d) IHC for AE1/AE3, ×250.

A 69-year-old man had a history of cataract surgery OD complicated by capsular rupture with anterior chamber intraocular lens (ACIOL) placement. The eye required subsequent pars plana vitrectomy with removal of retained lens fragments and ACIOL repositioning. Eighteen months after the initial surgery, his best corrected visual acuity was 20/40, but intraocular pressure (IOP) fluctuated up to 49 mmHg, and slit lamp examination revealed an area of apparent EDG involving 50% of the temporal cornea, with extensive anterior chamber angle closure (Figure 2).

The patient consented to diagnostic anterior chamber tap and two intracameral 5FU injections. In the operative suite, an anterior chamber tap with scraping of the epithelial membrane was performed, and a 30-gauge needle was used to deliver a mixture of 5FU 1000 mcg/0.1 mL and 0.1 mL viscoelastic (Amvisc, Bausch & Lomb, Rochester, NY) into the anterior chamber. After optimizing the ocular surface with treatments for the patient's worsened blepharitis and dry eye syndrome, a second injection of 5FU at 500 mcg/0.1 mL mixed with 0.1 mL viscoelastic was performed 3 weeks later. Anterior chamber paracentesis was performed at the beginning of the procedure and a pars plana tap at the end of the procedure to ensure normal IOP without disturbing the 5FU-viscoelastic mixture. Cytology of the anterior chamber aspirate showed epithelial squamous cells and nucleated eosinophilic cells, consistent with the diagnosis of EDG. Two months later, a glaucoma drainage device was implanted for angle-closure glaucoma. Four months after the 5FU injections, there was no clinical evidence of EDG, but he did require DSAEK for endothelial decompensation with vision declining to 20/400. Histopathologic analysis of the host specimen demonstrated rare epithelial cells that exhibited immunoreactivity for cytokeratin marker AE1/AE3, consistent with residual EDG (Figure 2).

Five months postoperatively, the patient remained stable with visual acuity of 20/50 and a clear graft without recurrent EDG.

## 4. Discussion

These reports describe cases of EDG with histopathologic and immunohistochemical clinicopathologic correlation following treatment with intracameral 5FU. These cases illustrate as well the effective collaboration of clinician and pathologist in EDG diagnosis via in-office endothelial scraping with aspiration of cell-laden aqueous.

In both cases, there was an early positive response to 5FU, with clinical evidence of regression of EDG and a favorable long-term result with minimal or no further destructive ablative therapy. This response suggests that the drug was indeed effective in decreasing the EDG cell burden, but its persistence/recurrence suggests that not all viable cells were eradicated, similar to the scenario often seen clinically with chemotherapy in the oncology arena. 5FU affects only cells that are actively proliferating; using more than one dose attempts to retarget temporarily immune cells in rest phase which may later activate and grow after the drug has cleared. We used two sequential injections of 1000 mcg 5FU in the first case and 1000 mcg followed by 500 mcg in the second case; previous reports described 40 to 1000 mcg doses, some with sequential injections [10–14]. In Case 1, it is possible that with air injection and manipulation some of the drug was lost through the paracentesis site. The second case employed needlesticks alone and developed no clinical evidence of persistence/recurrence, although some cellular presence was seen on histopathologic examination. Additionally, using a 5FU-viscoelastic mixture to fill the entire anterior chamber without admixed air may be preferable [12].

In both cases, this minimal intracameral intervention, followed by a single further procedure to rehabilitate the corneal endothelium, combined in Case 1 with localized cryotherapy only and in Case 2 with a tube shunt for IOP control, resulted in good clinical outcomes. Pharmacologically mediated decrease in the EDG proliferative cell burden and/or its activity apparently turned the clinical tide for these eyes.

Several previous reports also described an initial positive response to 5FU therapy, and longer-term outcomes at 5 to 6 months ranged from eradication of EDG [11–13] to recurrent EDG and corneal decompensation [10]. Complications of 5FU injection may include epithelial defect and possibly corneal decompensation. With multiple prior ocular surgeries and history of EDG that may have been more extensive than initially clinically evident, it is difficult to attribute endothelial failure to 5FU toxicity alone. Nonetheless, the close temporal relationship between the injections and the development of corneal edema suggests a toxic effect. Fortunately, endothelial dysfunction is more amenable to treatment than EDG, and both patients did well with subsequent corneal transplantation.

EDG remains an uncommon but potentially devastating complication of intraocular surgery. Intracameral antimetabolite therapy using an agent such as 5FU represents a potentially viable alternative to aggressive surgical intervention. Reports of initial EDG stabilization and regression following injection are promising, and improved long-term outcome is possible, although sequential corneal rehabilitation may be needed. The challenge going forward is to determine an optimal dose regimen and method of administration to ensure long-term success.
